# Spindle Epithelial Tumor of Thymus‐like Differentiation (SETTLE) in a 3‐year‐old African girl

**DOI:** 10.1002/ccr3.2163

**Published:** 2019-04-25

**Authors:** Olakayode O. Ogundoyin, Gabriel Ogun, Abideen Oluwasola, Tamaldeen A. Junaid

**Affiliations:** ^1^ Department of Surgery, College of Medicine University of Ibadan Ibadan Nigeria; ^2^ Department of Pathology, College of Medicine University of Ibadan Ibadan Nigeria; ^3^ Department of Pathology, Faculty of Medicine Kuwait University Kuwait City Kuwait

**Keywords:** ectopic, spindle epithelial tumor with thymus‐like differentiation, thymus, thyroid, tumor

## Abstract

Spindle epithelial tumor with thymus‐like differentiation (SETTLE) is a rare tumor of the thyroid gland occurring in children and young adults. This report presents SETTLE as a tumor that can also affect young children (under fives). Although majority of the reports were in Caucasians, the tumor can affect all races.

## 1 INTRODUCTION

Spindle epithelial tumor with thymus‐like differentiation (SETTLE) is an uncommon thyroid tumor of pediatric age group and young adults.^1^ The tumors often develop from ectopic thymus tissue and sometimes from remnants of branchial pouches that has the ability to develop along the thymic line.^2^ Histologically, the tumor is lobulated with biphasic cells consisting of spindle‐shaped epithelial cells that merge into glandular cells.^3^ It is often malignant but the tumors occurring in children may be benign.^4^ The youngest patient reported in the literature was 2 years old,^1^ we present the first case of SETTLE in a 3‐year‐old African girl who has had six years of follow‐up with no recurrence.

## 2 CASE REPORT

A 3‐year‐old girl presented with a painless and progressively increasing lateral neck mass since birth. Examination revealed a nontender, mobile, and multinodular left‐sided neck mass measuring 8 cm × 6 cm in dimension and there were no cervical or supraclavicular lymphadenopathies.

**Figure 1:**
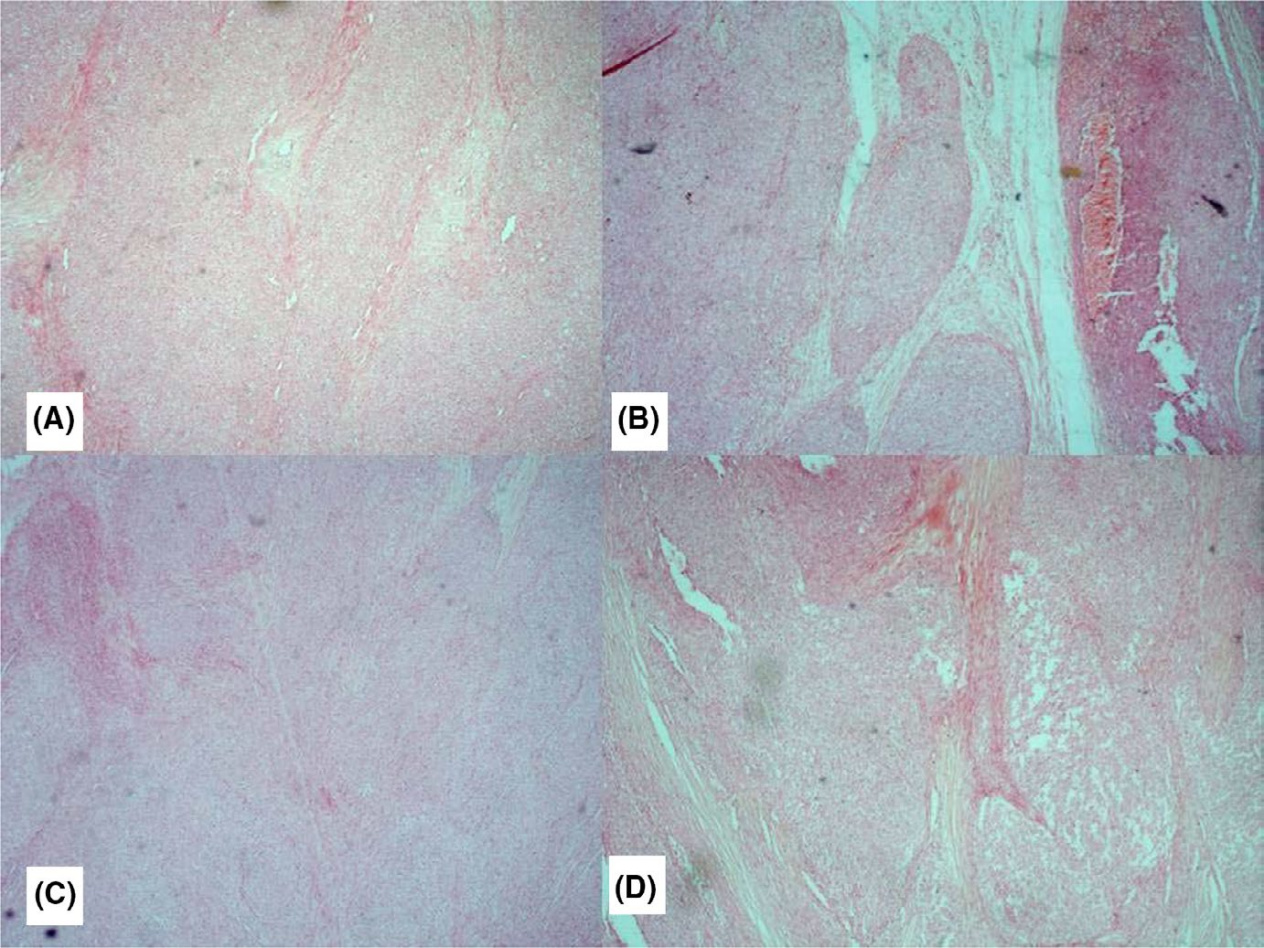
A‐D, Highly cellular tumor with proliferating spindle and polygonal (epithelial) cells occurring predominantly in lobulated and fasciculated patterns (H&E ×40)

**Figure 2:**
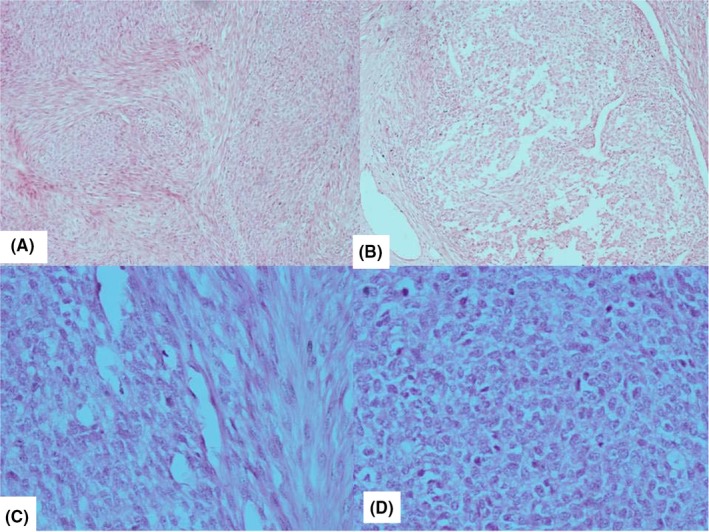
A‐C, The spindle cell component had hyperchromatic spindle nuclei with scanty to moderate eosinophilic cytoplasm (A&B–H&E ×200). D: The polygonal cells exhibited large vesicular nuclei (C&D–H&E ×400)

**Figure 3:**
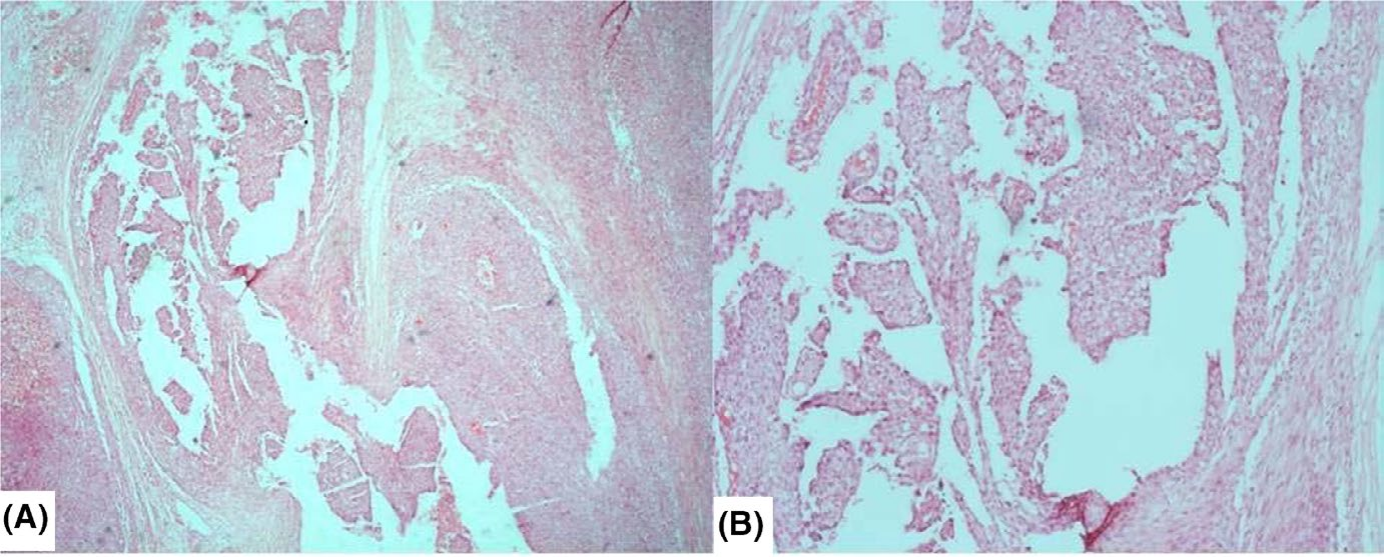
Cystic spaces lined by epithelial cells and containing irregular papillary structures (A–H&Ex100, B–H&E ×200)

Thyroid function test was normal while ultrasound of the neck revealed a solid lesion mainly on the upper pole of the left lobe of the thyroid gland. Fine needle aspiration cytology was suspicious of malignancy. Plain radiograph of the neck showed deviation of the trachea to the right side. At surgery, the left lobe of the gland was involved and a left lobectomy was done with no adjuvant therapy. Gross (macroscopic) examination of the tumor showed a nodular mass measuring 6 cm × 4 cm × 4 cm and weighing 35 g. Cut sections revealed a tan colored lobulated tumor, firm in consistency, and disposed in whorled appearance. Focal areas of cystic spaces were seen. The tumor was highly cellular with proliferating spindle and polygonal (epithelial) cells occurring predominantly in lobulated and fasciculated patterns (Figure 1). The spindle cell component had hyperchromatic oval nuclei with scanty to moderate eosinophilic cytoplasm (Figure 2A‐C), whereas the polygonal cells exhibited large vesicular nuclei. (Figure 2D) Foci of cystic spaces lined by epithelia cells that were disposed in irregular papillary patterns were also seen. (Figure 3) There was no area of necrosis. The resection margin was free of tumor but has residual unremarkable thyroid tissue. Based on initial hematoxylin and eosin sections, the differential diagnosis considered were solitary fibrous tumor, a peripheral nerve sheath tumor, and hyalinizing trabecular tumor.

Immunohistochemical study showed that both the spindle and polygonal cells were positive for pan cytokeratin (AE1/AE3), galectin‐3, and HBME but were negative for CEA, S‐100, CD 31, CD 34, chromogranin, calcitonin, p53, and CD117. Cytoplasmic positivity for smooth muscle actin (SMA) was seen in few of the spindle cells while about 5% of the tumor cells were Ki‐67 nuclear positive suggestive of low proliferative index. The final diagnosis of spindle epithelial tumor with thymus‐like differentiation (SETTLE) was made based on the histopathologic features and immunohistochemistry. Postoperatively, the recovery was good. She was discharged and followed up for about six years with no recurrence.

## 3 DISCUSSION

Spindle epithelial tumor with thymus‐like differentiation (SETTLE) is an uncommon tumor arising from the thyroid gland but yet to be well characterized^1^ and is believed to be a derivative of branchial pouch remnants or foci of ectopic thymus.^5^ This tumor had been reported variously in the past as thyroid spindle cell tumor, malignant teratoma of the thyroid gland, or thymoma of the thyroid gland.^4^ It was later reviewed and described as a cervical tumor derived from ectopic thymus or remnants of branchial pouch that retained their ability to differentiate along thymic lines.^2^, ^6^ It predominantly occurs in young people especially children, adolescents, and young adults but may be found in the middle age as it has been seen in patients aged 4‐59 years.

The tumor has a slight male preponderance than female with sex ratio of 1.5:1.^3^ Metastatic spread via the bloodstream is often delayed making it to be described as a low‐grade malignant tumor.^6^

Macroscopically, SETTLE appears encapsulated or partially circumscribed; it is firm, bosselated grayish white with an undefined whorled cut surface that may be lobular and cystic.^4^ The tumor is predominantly made up of spindle cells forming fascicles, which merge into focal glandular structures taking the form of tubules, papillae, and cystic spaces lined by respiratory‐type or cuboidal to columnar epithelium.^6^ It has a varying degree of cellularity displaying different patterns such as focal areas of herringbone, rosette‐like, palisading or papillary structures and sometimes the cellularity may be displayed in the periphery or adjacent to the fibrous bands.^7^ These characteristic gross and microscopic features were evident in our patient.

SETTLE has atypical clinical symptoms and signs and this makes preoperative diagnosis difficult as depicted from the review of previous cases.^8^, ^9^ A review of the literature showed 41 reported cases of SETTLE^10^ with the usual presentation of a painless thyroid or neck mass of either a short or long duration depicting the generally slow growth of the tumor.^11^

There is wide variation in the duration of clinical symptoms before the diagnosis is made with the range being 3 weeks^12^ to more than 10 years.^5^, ^9^, ^13^, ^14^ The tumor is located in the right lobe of the thyroid gland in most patients, occasionally the left lobe may be involved and sometimes both lobes of the gland.^9^ Rapid growth of the tumor leading to tracheal compression has been reported.^2^ The patient, however, presented with deviation of the tracheal to the right side with no demonstrable lymphadenopathy. Preoperative diagnosis could not be made in most of the previously reported cases and the diagnosis was delayed in three cases where autoimmune thyroiditis was suspected because of a confusing clinical and ultrasonographic picture.^2^, ^15^ Fine needle aspiration is often inconclusive 8, 9 but may arouse a suspicion of malignancy as in our patient. The relatively slow growth of the tumor makes the majority of reported cases amenable to surgical excision alone.^9^, ^11^ Surgical excision may range from nodulectomy, lobectomy to subtotal thyroidectomy. Adjuvant chemotherapy and radiotherapy have been used in cases of widespread metastasis.^4^

Although SETTLE exhibits delayed metastasis, it must be treated as a potentially malignant tumor as it can metastasize several years after surgical resection with the lungs as one of the commonest sites of metastasis.^4^, ^8^

In conclusion, we report here the first case of SETTLE in Nigeria and most probably from sub‐Saharan Africa. The clinical presentation and behavior were typical and similar to the previously reported cases. Early diagnosis and surgical resection of the tumor with meticulous long‐term follow‐up of the patients is key to ensuring a good prognosis.

## CONFLICT OF INTEREST

We also want to declare that there are no conflicts of interest in the production of this manuscript and the work was solely funded by the authors.

## AUTHOR CONTRIBUTION

OOO: Has made substantial contributions to conception and design and acquisition of data; has been involved in drafting the manuscript and revising it critically for important intellectual content. Has given final approval of the version to be published and has agreed to be accountable for all aspects of the work in ensuring that questions related to the accuracy or integrity of any part of the work are appropriately investigated and resolved. GOO: Has been involved in analysis and interpretation of data; drafting the manuscript and revising it critically for important intellectual content. Has given final approval of the version to be published and has agreed to be accountable for all aspects of the work in ensuring that questions related to the accuracy or integrity of any part of the work are appropriately investigated and resolved. AOO: Has made substantial contributions to conception and design; acquisition of data; analysis and interpretation of data. Has been involved in drafting the manuscript and revising it critically for important intellectual content and has given final approval of the version to be published. TAJ: Has been involved in analysis and interpretation of data; drafting the manuscript and revising it critically for important intellectual content. Has given final approval of the version to be published and has agreed to be accountable for all aspects of the work in ensuring that questions related to the accuracy or integrity of any part of the work are appropriately investigated and resolved.
